# NP-guide: a portable projection-based navigation system for neurosurgery and beyond

**DOI:** 10.3389/fneur.2025.1691434

**Published:** 2025-11-03

**Authors:** Zhongjie Shi, Xin Gao, Sifang Chen, Deyong Xiao, Zhangyu Li, Xiaojun Li, Yilong Peng, Jiajia Yu, Zhanxiang Wang

**Affiliations:** ^1^Department of Neurosurgery, The First Affiliated Hospital of Xiamen University, School of Medicine, Xiamen University, Xiamen, China; ^2^National Institute for Data Science in Health and Medicine, Xiamen University, Xiamen, China; ^3^Xiamen Neurosurgical Quality Control Center, Xiamen, China; ^4^Department of Neurosurgery, Jiangmen Central Hospital, Jiangmen, China; ^5^College of Electronics and Information Engineering, Wuyi University, Jiangmen, China

**Keywords:** neurosurgery, NP-guide, augmented reality, surgical navigation, proof-of-concept study

## Abstract

**Background:**

Stereotactic systems and various robot-assisted navigation platforms in neurosurgery have enabled high-precision localization. However, these systems, while highly accurate, are expensive, technically demanding, and procedurally complex, making them less practical for routine use. This study introduced and evaluated the Navigation and Projection Guide (NP-Guide), a projection-based augmented reality (AR) system designed to provide a portable and accessible solution for surgical navigation.

**Methods:**

NP-Guide, a mobile application, projects patient imaging data and three-dimensional (3D) reconstructions onto the patient’s head surface to assist with localization. This proof-of-concept study prospectively enrolled 52 neurosurgical patients, randomized to the NP-Guide group (*n* = 27) or the freehand localization group (*n* = 25). Two physicians with different training backgrounds performed the procedures. Localization error and operating time were measured using a commercial optical navigation system (ONS). Bland–Altman analysis was applied to assess inter-operator agreement, and learning curves were generated to evaluate proficiency.

**Results:**

Baseline characteristics were comparable (all *p* > 0.05). In the NP-Guide group, mean localization error was 4.1 ± 2.1 mm for Physician A and 3.4 ± 1.8 mm for Physician B, with mean times of 1.2 ± 0.5 min and 1.1 ± 0.4 min, respectively. Compared with freehand localization, NP-Guide significantly improved the accuracy and efficiency (all *p* < 0.001). Bland–Altman analysis demonstrated good inter-operator agreement; no significant difference was observed (*p* = 0.25). Learning curves showed that operating times plateaued at approximately 1 min after about 15 cases.

**Conclusion:**

The NP-Guide demonstrated accurate, efficient, and reproducible projection-based localization in this proof-of-concept study. Its portability, low cost, and ease of use suggest potential value, particularly in resource-limited settings. However, these findings should be interpreted as preliminary, and further phantom experiments and multicenter clinical studies are required before widespread adoption in routine practice.

## Introduction

Common neurosurgical procedures include surgery for intracerebral hemorrhage (ICH), brain tumor resection, external ventricular drainage (EVD), and traumatic brain injury (TBI) (1–3). Regardless of the type of procedure, systematic and accurate preoperative planning is essential to ensure surgical safety and improve outcomes. The ability to reproduce the preoperative plan is directly linked to both safety and efficacy.

Preoperative planning relies mainly on imaging data that delineate the lesion and anatomical structures, together with the surgeon’s initial design of the surgical approach. However, in the absence of high-precision navigation systems in the operating room (OR), surgeons must rely on their anatomical knowledge, training, and clinical experience to translate two-dimensional (2D) images into three-dimensional (3D) anatomy. This approach is highly experience-dependent and prone to errors, especially in complex cases or when anatomical landmarks are unclear (4, 5).

The introduction of neuro-navigation and surgical robots has significantly enhanced surgical precision, enabling guidance at the millimeter or even sub-millimeter level (6–8). However, these systems are expensive, bulky, and require complex workflows, which limits their use in smaller hospitals or resource-constrained regions (9, 10). Even in well-equipped centers, experienced neurosurgeons often prefer freehand localization for certain procedures involving large lesions with obvious anatomical landmarks (e.g., basal ganglia hemorrhage). In such cases, the precision advantage of navigation is relatively small, whereas the time required for the system setup may delay surgery, especially in emergency settings (11, 12).

For routine neurosurgical procedures that do not require millimeter-level accuracy but still require reliable localization, such as ICH, EVD, or convexity meningioma resection, there is a clear need for a low-cost, user-friendly, and rapidly deployable tool (13). Such a tool should reproduce preoperative planning at the bedside, reduce reliance on spatial imaging, and minimize additional intraoperative time.

In this study, we developed a Navigation and Projection Guide (NP-Guide), a mobile application based on augmented reality (AR). Unlike existing AR navigation systems, which often require head-mounted displays, fiducial registration, or complex hardware integration, NP-Guide adopts a projection-based approach that directly overlays preoperative images onto the patient’s scalp using a mobile device, thereby avoiding elaborate calibration and shortening setup time. By providing intuitive visual guidance that can be readily interpreted regardless of the surgeon’s level of experience, NP-Guide reduces operator dependency inherent to freehand localization. Its lightweight design and rapid deployability also facilitate seamless integration into the surgical workflow, particularly in emergency scenarios where conventional navigation may be impractical. This study aimed to provide a proof-of-concept evaluation of NP-Guide, comparing it with freehand localization in neurosurgical cases, to explore its feasibility and accuracy as a practical and low-cost localization tool rather than a definitive clinical validation.

## Materials and methods

### Program design

The NP-Guide was developed using the Unity engine and runs on Android devices without requiring internet access. At the first launch, the program requests permission to access the camera and the local files. The camera provides real-time video as the display background, whereas local files are used to load projection images for localization. The current implementation is compatible with Android-based smartphones and tablets, which must support camera access and basic graphics rendering. No additional external hardware or specialized sensors are required, and there are no strict performance constraints beyond the standard capabilities of modern Android devices.

The application was implemented with a modular architecture comprising three core components: an image import module for loading preprocessed projection images generated from medical imaging data, a manual registration module that supports affine transformations and a projection rendering module that overlays the processed image onto the live camera feed in real time. This modular design allows each component to be optimized independently while ensuring overall robustness and stability during intraoperative use.

In this study, NP-Guide was deployed on a Huawei Mate P70 Pro smartphone with an OLED display (2,844 × 1,260 pixels resolution, adaptive refresh rate up to 120 Hz). The commercial navigation system used as the reference standard was the Brainlab navigation system (Brainlab AG, Germany). This setup was used solely for proof-of-concept validation; further optimization and cross-device testing will be required before clinical adoption.

**Figure 1:**
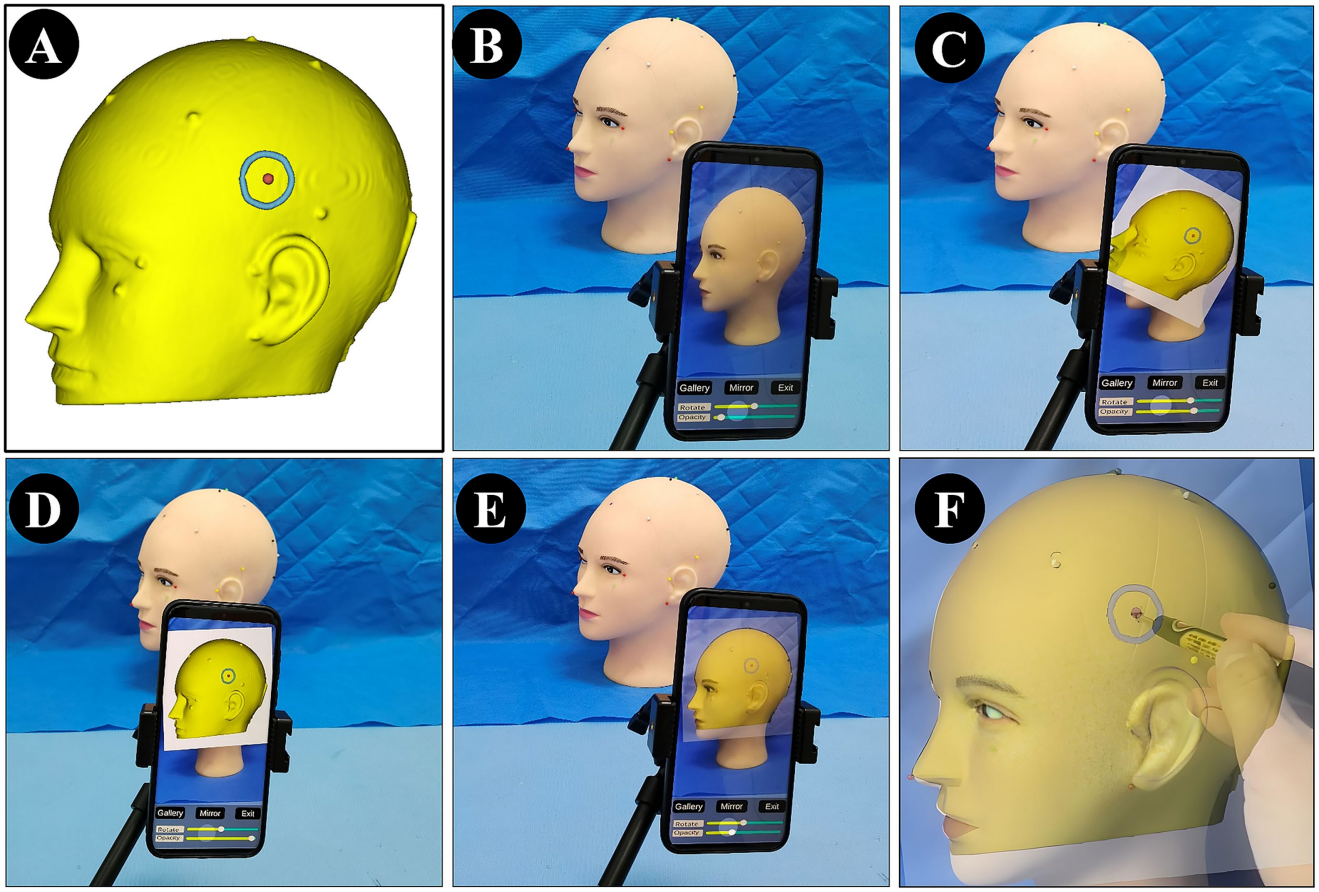
Workflow of NP-Guide localization using a simulated head model. **(A)** Yellow indicates the skin model; green shows the projected boundary of the lesion on the scalp; the red dot marks the projected lesion center (target point) on the scalp. **(B)** NP-Guide automatically activates the camera as the background. **(C,D)** Import the image for localization and adjust its angle and position through rotation and translation. **(E,F)** Adjust image transparency to match the contour of the head model **(E)**, and mark the lesion site **(F)**.

To facilitate rapid alignment between the image and the patient’s head, the system includes rotation, scaling, translation, and transparency adjustment functions. After positioning the mobile device at an appropriate angle, users can complete the matching process without repeatedly moving the device, which improves both efficiency and stability. It should be emphasized that as a rough auxiliary localization approach, NP-Guide does not employ any automated registration algorithm. Instead, alignment is achieved manually through affine adjustments (rotation, scaling, and translation) and by positioning the mobile device at an appropriate angle until the projected image adequately overlaps with the patient’s scalp contour. This design choice was intentional: automated registration would require additional algorithm development and reliable intraoperative segmentation of patient-specific head contours, which increases computational demands and hardware requirements. In contrast, the manual adjustment process is straightforward, efficient, and consistent with the exploratory, proof-of-concept nature of NP-Guide. Once the user observes that the contours are adequately matched, the registration process is considered complete. The workflow of NP-Guide using a simulated head model is shown in Figure 1.

### Patients

Patients with intracranial space-occupying lesions who underwent neurosurgical procedures at our institution between August 2024 and June 2025 were enrolled in this proof-of-concept feasibility study, which was approved by the institutional ethics committee. Informed consent was obtained from all participants.

#### Inclusion criteria

Patients older than 18 years; intracranial lesions confirmed by imaging, with clearly defined margins located supratentorially, or requiring EVD due to ICH or hydrocephalus. All the patients and their families agreed to undergo surgery and provided signed informed consent.

#### Exclusion criteria

Multiple lesions, reoperation with obvious scalp scars, pregnancy, or severe systemic diseases such as cardiac disorders.

### Data processing

The data were processed using the open-source medical imaging software 3D Slicer (14). All patients underwent cranial CT (slice thickness ≤ 1 mm; other scan parameters were not standardized). Patients with tumors additionally underwent MRI as clinically indicated. Imaging data were exported in the DICOM format (15).

Processing was performed collaboratively by a medical PhD with experience in 3D Slicer and two attending neurosurgeons with different levels of experience (Physician A: junior attending; Physician B: senior attending). For multimodal images, registration was performed using the *Elastix* module in the 3D Slicer. A scalp incision was made based on the lesion location. The Segment Editor module was used to outline the incision and mark its center point (Point-C). If artifacts made the scalp contour unclear, anatomical landmarks on the skin surface (e.g., nasal tip, lateral canthus, tragus) were used as references, or the dataset was rescanned when necessary. The Mask Volume tool was then applied to fill the region with high-intensity values. After processing, resampling was performed using the *Create a DICOM Series* module, and new DICOM data were exported for use in the intraoperative optical navigation system (ONS).

**Figure 2:**
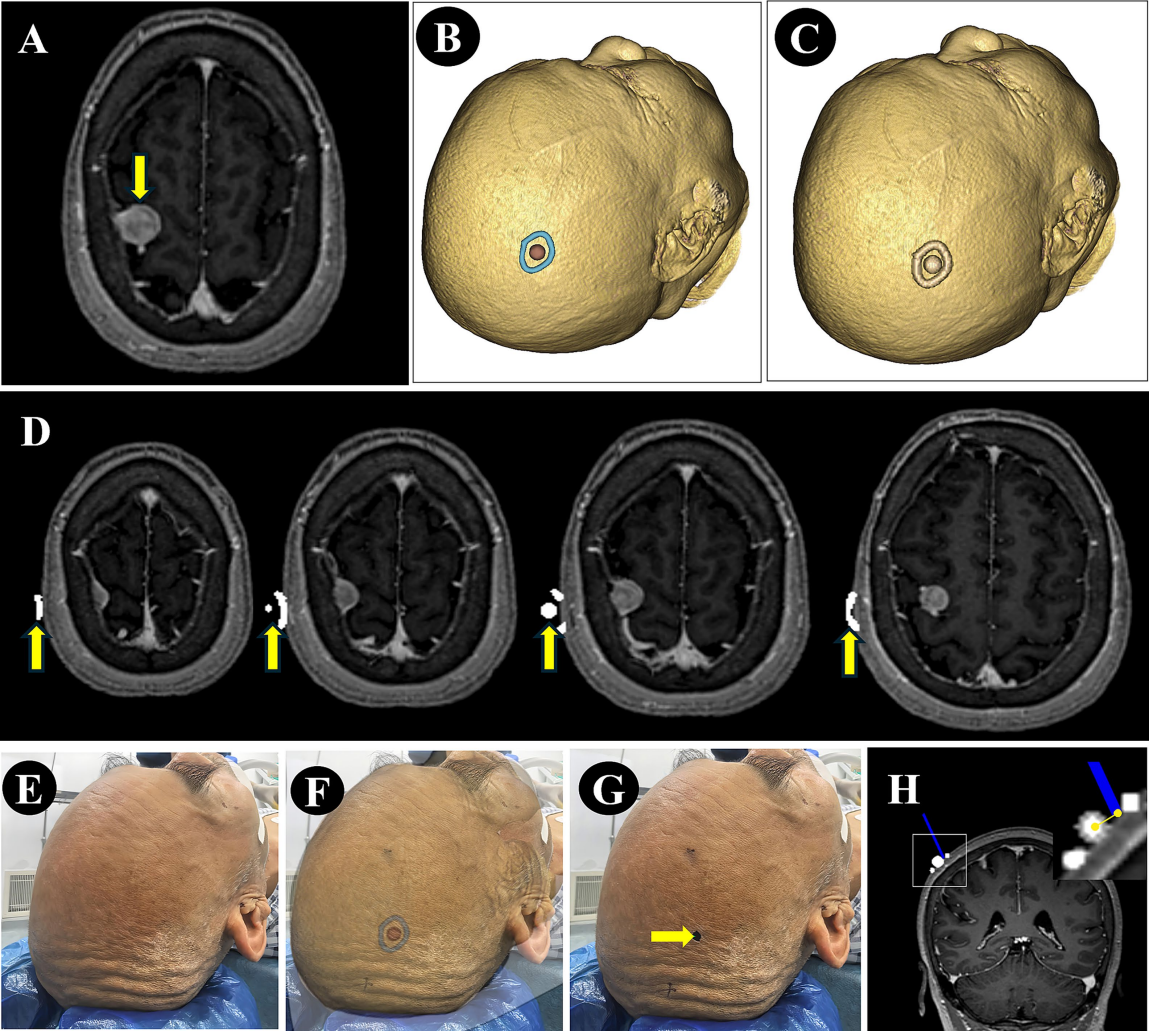
Image processing and error measurement. **(A)** A right parietal lesion was shown on preoperative MRI (purple arrow). **(B)** The lesion boundary (blue) and center point (red) were reconstructed from preoperative imaging and the view was saved for NP-Guide localization. **(C)** The lesion boundary and center point were filled in grayscale and fused with the original image. **(D)** The fused image was resampled and exported in DICOM format, containing the lesion boundary and center point (yellow arrow). **(E–G)** The patient’s head was fixed **(E)**, the NP-Guide projection was applied **(F)**, and the lesion center was marked on the scalp (green arrow, **G**). **(H)** An optical navigation probe (blue) was placed on the marked point, and the ONS was used to measure the distance between the probe tip and the true lesion center.

For patients in the NP-Guide group, after exporting the DICOM data, the 3D view was further adjusted to display the lesion contour and its center. This view was then captured using a mobile device running NP-Guide to enable projection-based localization during the surgery. The workflow for the data processing and measurement is shown in Figure 2.

### Accuracy evaluation

After the induction of general anesthesia, the patient’s head was fixed in the planned surgical position and the incision area was fully exposed. Registration was performed by the same surgeon (Physician C) following the standard ONS workflow. Patients had been randomly assigned preoperatively to the NP-Guide group or the freehand group using a computer-generated random number table. The allocation sequence was generated and kept by an independent research assistant who was not involved in patient enrollment, surgery, or outcome assessment.

Within each group, both Physician A and Physician B independently performed localization using the assigned method. Before patient enrollment, both physicians underwent a structured training session on NP-Guide, which included a 30-min tutorial on system functions and at least 10 practice localization attempts on a simulated head model.

The order of physician participation was randomized using sealed opaque envelopes to avoid selection bias. For each localization, the operating physician determined the lesion center (Point-C) and marked the point on the scalp. Physician C then placed the tip of the optical navigation probe at the marked point. The Euclidean distance between the probe tip and the true lesion center (Point-C), as displayed by the navigation system, was defined as the target registration error (TRE) for that physician and method.

After each measurement, the scalp mark was erased before the second physician repeated localization using the same method, blinded to the first physician’s result. An independent observer, blinded to both group allocation and operator identity, simultaneously recorded the localization time and error.

**Figure 3:**
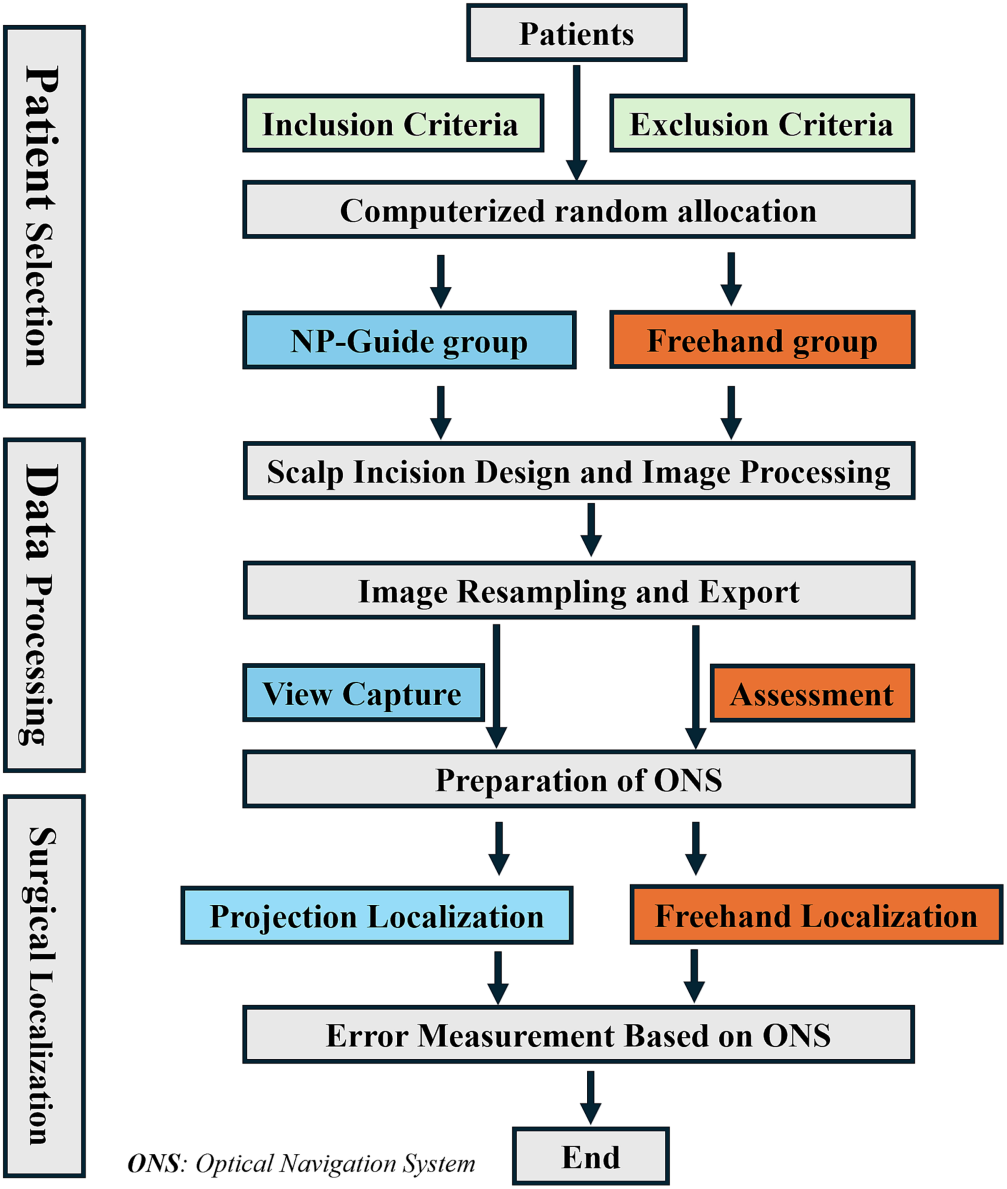
Study workflow. The entire process was conducted from patient enrollment to the completion of measurement using ONS.

In this study, the optical navigation system was uniformly applied as an the intermediate measurement tool to eliminate registration errors between systems, reduce subjective measurement variability, and ensure comparability of the localization accuracy. An independent observer simultaneously recorded both the localization time and error for each method (Figure 3).

It should be noted that in this study, the evaluation was performed in parallel with standard navigation to generate preliminary accuracy data only. Localization using NP-Guide or freehand was conducted solely for data recording and accuracy assessment, without interfering with the routine surgical workflow. The final surgical plan for each patient was determined independently by the operating surgeon, based on personal experience and the results of the commercial ONS.

### Sample size estimation and statistical analysis

The sample size calculation was primarily based on the primary endpoint, that is, the localization error. According to the preliminary pilot data, the expected mean ± SD localization error was 5 ± 4 mm in the NP-Guide group and 15 ± 10 mm in the freehand group. Assuming unequal variances, the sample size was estimated using Welch’s t test with a significance level of *α* = 0.05, and a statistical power of 0.90. The calculation indicated that at least 15 patients were required per group. To account for potential dropouts and missing data, at least 18 patients per group were enrolled. The sample size calculation was performed using Python version 3.10 with the statsmodels library and verified with the exact power method for Welch’s t test. This study design also adhered to the GRACE (Good Research for Comparative Effectiveness) principles (https://www.graceprinciples.com/index.html) and followed the GCP (Good Clinical Practice) guidelines (https://www.ema.europa.eu/en/ich-e6-good-clinical-practice-scientific-guideline) to ensure data integrity and reporting transparency [cf. Heller R, Krieger A, Rosset S. Optimal multiple testing and design in clinical trials. Biometrics. 2023; 79 (3):1908–1919].

All statistical analyses were performed using SPSS version 26.0 (IBM Corp., Armonk, NY, USA). Quantitative data are expressed as mean ± standard deviation (SD). Between-group comparisons of localization time and localization error were conducted using the Mann–Whitney U test. Comparison of localization errors between physicians was performed using the Wilcoxon signed-rank test. Categorical variables were presented as counts, and group differences were assessed using the chi-square test. Statistical significance was defined as a two-tailed *p*-value < 0.05. All statistical analyses were exploratory in nature, consistent with the proof-of-concept design of the study.

## Results

A total of 52 patients were enrolled in this feasibility study. All patients completed the planned workflow without missing data or dropouts, and all measurements were available for analysis. The NP-Guide group included 27 patients (16 males and 11 females; mean age 51.2 ± 8.6 years, range 29–74 years), consisting of 12 cases of intracerebral hemorrhage, 8 meningiomas, 4 gliomas, and 3 cases of hydrocephalus. The Freehand group included 25 patients (15 males and 10 females; mean age 49.5 ± 9.2 years, range 23–71 years), consisting of 10 cases of intracerebral hemorrhage, 9 meningiomas, 5 gliomas, and 1 case of hydrocephalus. There were no statistically significant differences between the two groups in baseline characteristics, including sex, age, and disease distribution (all *p* > 0.05), indicating good comparability.

**Figure 4:**
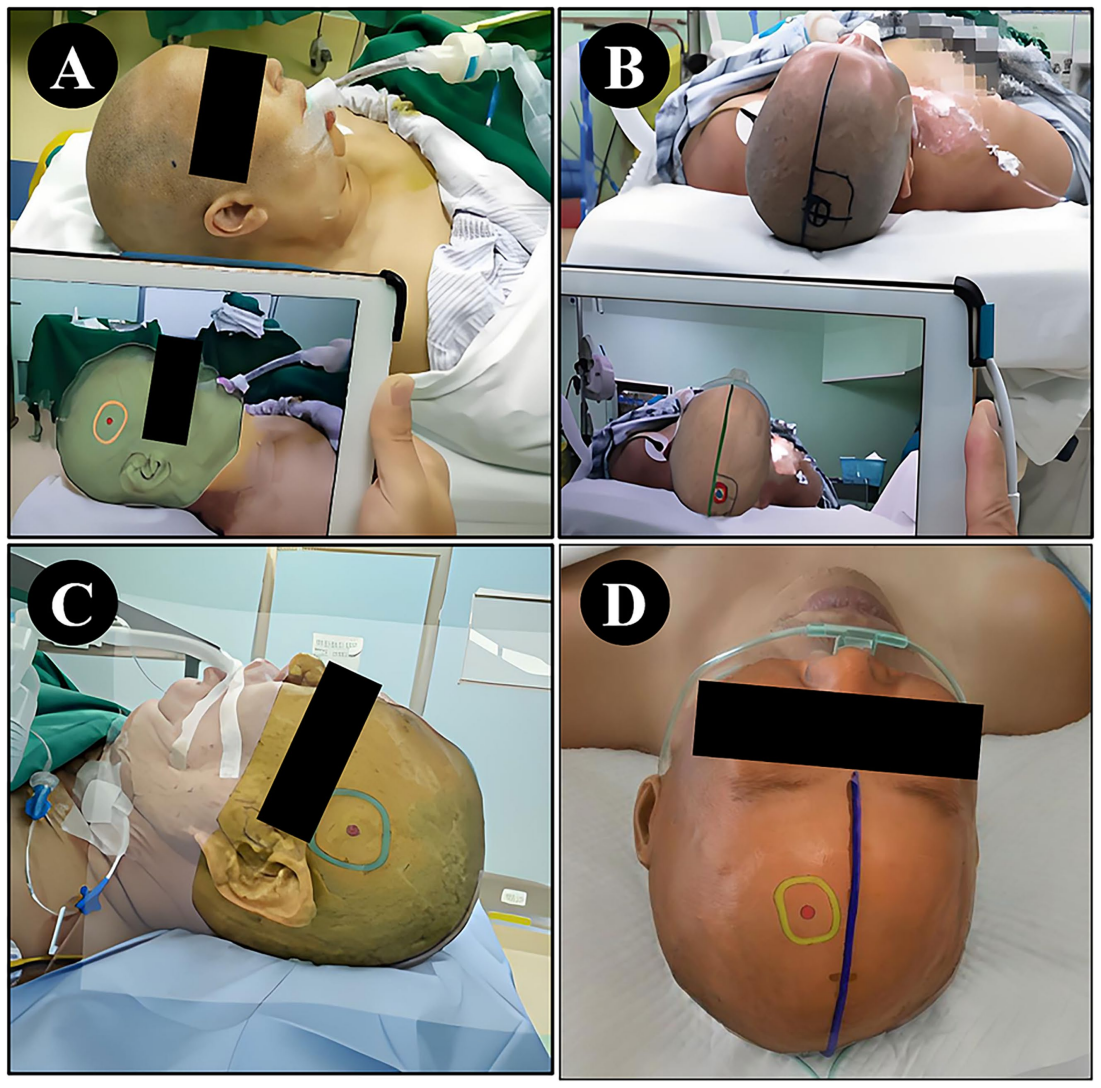
Localization using NP-Guide. **(A)** Localization of a right temporal lesion. **(B)** Localization of a right parietal lesion. **(C)** Localization of a left temporal lesion. **(D)** Localization of a left frontal lesion.

The NP-Guide functioned stably during use in this exploratory setting, with no startup failures or other system errors. Both physicians successfully performed preoperative localization at common surgical sites, including the temporal, frontal, and parietal regions (Figure 4).

Compared with freehand localization, Physician A and Physician B achieved significantly shorter localization times and higher localization accuracies when using NP-Guide (all *p* < 0.05, Table 1).

**Table 1:** Comparison of results for two physicians using different localization methods.

Doctor	Variable	Method	Mean ± SD	Range	95% CI	Statistic	*P*-value
Doctor A	Time (min)	NP-Guide	1.2 ± 0.5	0.6–2.3	1.0–1.4	*U* = 77.5	*P* < 0.001
		Freehand	2.2 ± 0.6	1.3–3.2	2.0–2.5		
	Error (mm)	NP-Guide	4.1 ± 2.1	1.0–8.0	3.3–5.0	*U* = 19.5	*P* < 0.001
		Freehand	19.1 ± 6.8	5.0–31.0	16.3–21.9		
Doctor B	Time (min)	NP-Guide	1.1 ± 0.4	0.6–2.0	0.9–1.3	*U* = 54.0	*P* < 0.001
		Freehand	2.0 ± 0.5	1.3–2.9	1.8–2.2		
	Error (mm)	NP-Guide	3.4 ± 1.8	1.0–7.0	2.7–4.2	*U* = 23.5	*P* < 0.001
		Freehand	14.1 ± 5.5	3.0–22.0	11.9–16.4		

**Figure 5:**
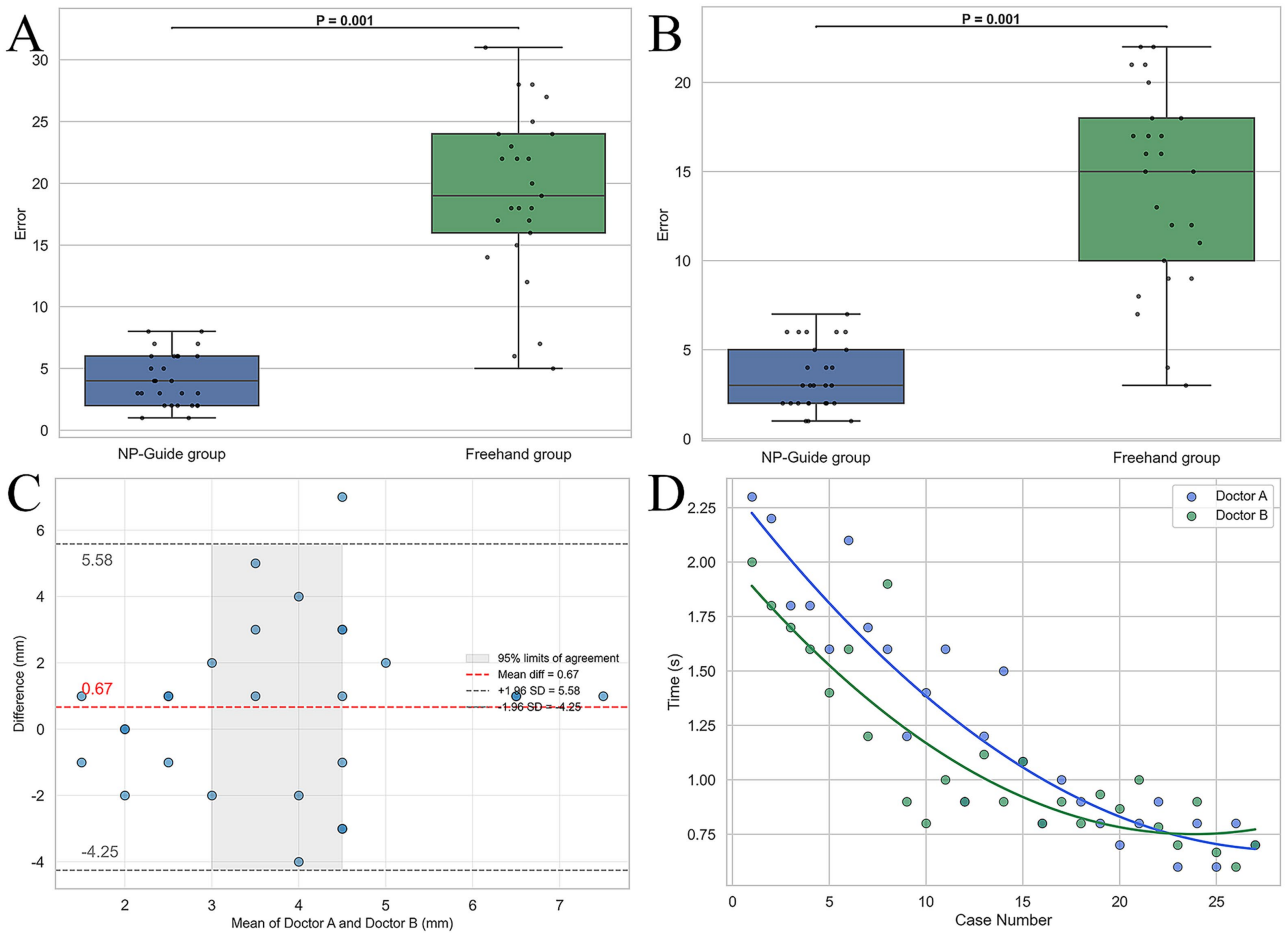
Statistical analysis of localization results for two physicians. **(A,B)** Boxplots of localization errors for Physician A and Physician B under NP-Guide and freehand localization. **(C)** Bland–Altman analysis of NP-Guide localization between the two physicians, showing good agreement. **(D)** Learning curves of NP-Guide localization for both physicians.

With NP-Guide localization, the mean error was 4.1 ± 2.1 mm for Physician A and 3.4 ± 1.8 mm for Physician B, with no statistically significant difference between them (*p* = 0.25). Under freehand localization, the mean errors were 19.1 ± 6.8 mm and 14.1 ± 5.5 mm, respectively, showing a significant difference (*p* = 0.03). Learning curve analysis further demonstrated that when approximately 15 cases had been completed with NP-Guide, the localization time gradually stabilized at approximately 1.0 min (Figure 5). These findings should be interpreted within the scope of a proof-of-concept study.

## Discussion

Neurosurgical localization generally involves both preoperative and intraoperative procedures. Preoperative localization relies mainly on imaging data combined with cranial or scalp anatomical landmarks to determine the extracranial projection of the lesion, thereby guiding the incision design and surgical approach planning. Intraoperative localization is achieved by direct anatomical identification or by using neuro-navigation, intraoperative imaging, and neurophysiological monitoring to confirm the lesion and its relationship with critical neurovascular structures, improving surgical precision and safety (16–18). However, institutions with limited resources or in clinical settings where millimeter-level precision is not required, localization often depends on the surgeon’s anatomical knowledge, training, and clinical experience.

### Advantages and preliminary performance of NP-guide

As a mobile application, NP-Guide is not restricted by location and can be used in operating rooms, wards, and other clinical settings. In this study, a skin-based 3D model matching method was used. In clinical practice, when imaging data cannot be exported, sagittal, coronal, or axial slices from the hospital imaging system can be used for rapid localization, ensuring flexibility across different environments. Further details of the NP-Guide operation are provided in Supplementary material 1 and Supplementary Video 1 to facilitate its clinical adoption.

Compared with established neuronavigation systems, NP-Guide offers several practical advantages. First, the system is low-cost, requiring only a standard Android smartphone or tablet, in contrast to commercial platforms that involve expensive optical tracking cameras, dedicated workstations, and proprietary software. Second, NP-Guide is highly portable and easy to use, with a workflow that can be completed within minutes without the need for fiducials, elaborate calibration, or integration with additional intraoperative hardware. Third, the learning curve is relatively short: both junior and senior physicians achieved stable performance after limited training on a simulated model, whereas conventional neuronavigation typically requires more extensive training and technical support. These attributes highlight NP-Guide’s potential value as an accessible alternative, particularly in resource-limited or emergency settings where traditional systems may be impractical.

Previous studies have explored the use of mobile projection tools. For example, the Sina APP projection method achieved a mean localization error of approximately 4.4 mm with an average time of 2.4 min, demonstrating the potential of mobile-based tools (19, 20). However, the Sina APP requires manual movement and rotation of the device to achieve alignment, which poses practical limitations. In contrast, NP-Guide allows direct image adjustment through rotation, scaling, and translation, while device stabilization with a stand prevents hand tremors from affecting the accuracy. Recent advances in mixed reality (MR) have shown promise in neurosurgery, enabling both preoperative and intraoperative localization with millimeter-level precision, which represents the future direction of surgical navigation (21–23). However, previous studies have confirmed that, some users may experience dizziness, visual fatigue, or neck discomfort when wearing head-mounted displays (HMDs), which may limit their widespread clinical adoption (24). Compared with such advanced systems, the NP-Guide emphasizes low cost and ease of use while maintaining accuracy, making it a promising low-cost alternative for exploratory use and potential future adoption after further validation. The NP-Guide may also serve as a lightweight front-end for future intelligent navigation systems that incorporate mixed reality and artificial intelligence.

This study further showed that Physician A exhibited significantly higher error than Physician B during freehand localization (19.1 ± 6.8 mm vs. 14.1 ± 5.5 mm, *p* = 0.03), indicating that freehand accuracy is closely related to operator experience. By contrast, with NP-Guide assistance, no significant difference was found between the two physicians (4.1 ± 2.1 mm vs. 3.4 ± 1.8 mm, *p* = 0.25), suggesting good generalizability and robustness of the tool in reducing operator variability. Moreover, both physicians achieved significantly lower errors with NP-Guide than with freehand localization (all *p* < 0.001), further suggesting its potential to improve accuracy and reduce inter-operator differences in this proof-of-concept setting.

### Intraoperative applications and potential

This study primarily validated NP-Guide for preoperative localization. While the underlying principle suggests potential intraoperative applicability, such use remains speculative at this stage. In theory, cortical vessels, sulci, and gyri could serve as intraoperative registration references, enabling integration of visible structures with preoperative reconstructions for real-time projection-based guidance. However, this approach assumes negligible brain shift, which represents a critical limitation (25). Future research should specifically evaluate the feasibility of NP-Guide in intraoperative settings and explore strategies to compensate for brain shift. Beyond neurosurgery, NP-Guide may also have cross-disciplinary potential, for example in dentistry, orthopedics, and thoracic surgery, where bony or surface landmarks can be used for registration. These applications are likewise speculative and require dedicated validation.

### Clinical implementation and practical insights

During clinical implementation, no adverse events such as skin injury or intraoperative interference were observed. Because NP-Guide operates on handheld Android devices, slight hand tremors can occur during image alignment and target marking, particularly when holding the device for extended periods. This may introduce small positional deviations and user fatigue. To improve stability, we recommend the use of a mobile device stand or fixed mount during clinical operation. This adjustment effectively eliminates hand-induced motion and ensures more consistent alignment between the projected image and the scalp contour. These practical insights will be integrated into future design iterations to enhance ergonomics and usability.

### Limitations

This study had several limitations. First, the sample size was small and from a single center, limiting generalizability. Lesion types were not stratified; however, given the small cohort, stratification would likely not yield meaningful differences. Second, the accuracy evaluation relied on a single ONS without cross-validation against multiple modalities, and no phantom model with known coordinates was used. The lack of phantom-based validation is a major limitation, as such experiments are essential for assessing absolute accuracy. Third, only two physicians with different levels of experience were included, which may not represent a broader population. Potential sources of bias should also be acknowledged. Patient selection bias may have influenced the findings, as only cases meeting specific inclusion criteria were enrolled, while complex or atypical lesions were excluded. Operator-related bias is possible, given that both physicians were from the same institution and underwent similar training. Finally, reliance on a single navigation system as the reference standard may introduce measurement bias.

From a technical perspective, several limitations should also be noted. Although NP-Guide provides rotation, scaling, and translation functions, these manual adjustments cannot fully compensate for the intrinsic parallax effect. Projection-based techniques require precise angular alignment between the projected image and the scalp contour. This issue is more pronounced for deep lesions, where minor surface misalignments may be magnified along the surgical trajectory. Moreover, NP-Guide does not account for intraoperative brain shift. Because the projection relies on preoperative images, tissue deformation during surgery would reduce accuracy. Future iterations could mitigate these issues through device stabilization mounts, automated or AI-assisted registration, and integration with intraoperative imaging modalities. Finally, the current localization approach requires full scalp preparation, which may limit applicability in female patients or procedures involving only partial exposure.

### Future directions

Future studies should involve larger, multicenter cohorts to validate NP-Guide and extend its application to challenging scenarios. Integration with advanced technologies, including MR, artificial intelligence, and computer vision–based real-time scalp contour extraction (e.g., OpenCV), may further enhance accuracy and functionality while preserving portability. Moreover, direct comparisons with commercial navigation systems using phantom or simulation models, along with cross-validation across multiple imaging modalities, will be essential for rigorous performance evaluation (26). Finally, systematic safety assessments and regulatory approval will be necessary steps toward broader clinical adoption.

## Conclusion

In summary, NP-Guide, as a mobile projection-based localization tool, demonstrated promising accuracy, efficiency, and usability in this study. Its low cost, portability, and ease of use highlight its potential value, particularly in resource-limited environments. However, as a proof-of-concept exploration, this system still requires further phantom experiments and multi-center studies to establish its reliability and broaden its translational applicability across disciplines.

### Software statement

NP-Guide is an open-access software designed for academic research and medical education. It has not obtained medical device certification or regulatory approval and must not be used for clinical diagnosis or therapeutic decision-making. Users are required to comply with applicable laws and regulations in their jurisdictions, and any risks or liabilities arising from their use remain the responsibility of the user. The authors and developers disclaim liability for use beyond research and educational purposes.

## Data Availability

The original contributions presented in the study are included in the article/Supplementary material, further inquiries can be directed to the corresponding authors.
