# Supplementary material for: NP-guide: a portable projection-based navigation system for neurosurgery and beyond
Source: Front Neurol. 2025 Nov 3;16:1691434. doi: 10.3389/fneur.2025.1691434 (PMC12620224; doi:10.3389/fneur.2025.1691434)
Supplement: Supplementary file 2 [file Presentation_1.pdf]

NP-Guide: Introduction and Usage

NP-Guide is an Android-based projection-assisted localization tool. In this document, we provide a detailed description of NP-Guide's image processing steps, workflow, and practical usage tips, with **Figure 1** illustrating the image processing workflow based on 3D Slicer, and **Table 1** presenting a comparison of the features of NP-Guide and conventional navigation systems.

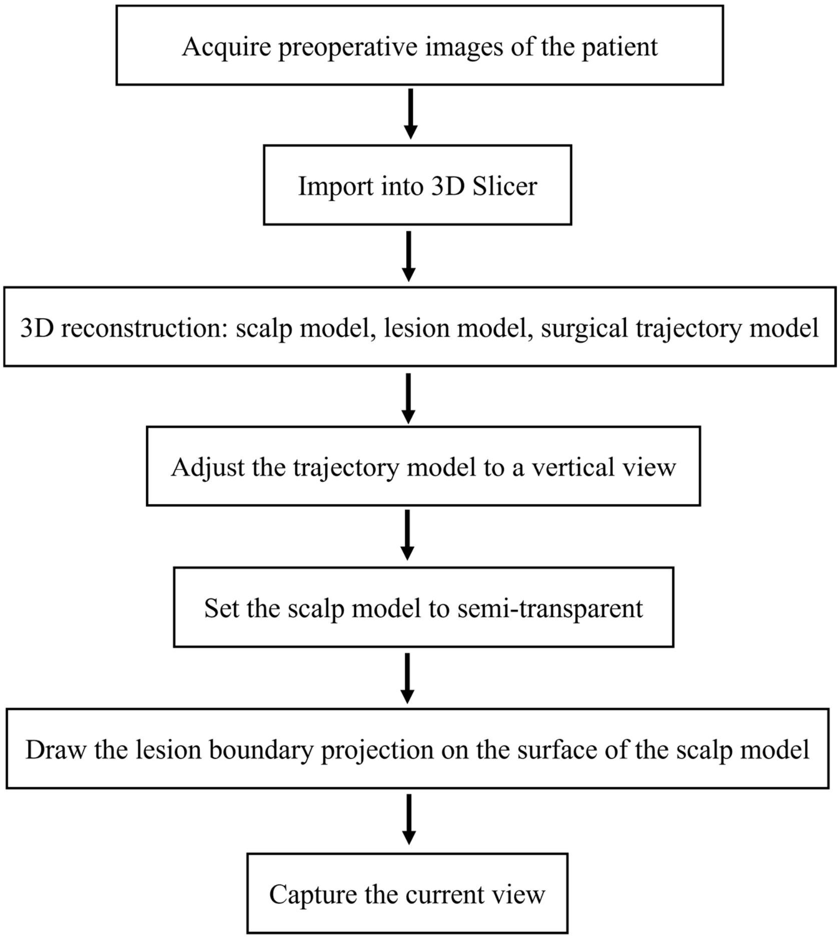

Figure 1. Workflow Diagram Based on NP-Guide and 3D Slicer

Table 1. Summary of the Comparison Between NP-Guide and Conventional Navigation.

Feature	NP-Guide (projection-based AR)	Conventional neuronavigation systems
Cost	Requires only a standard Android smartphone or tablet; low cost	Requires optical tracking cameras, dedicated workstation, and proprietary software; often >100,000 USD
Portability	Lightweight, mobile; usable in operating rooms, wards, or emergency settings	Bulky, fixed to the operating room; limited portability
Setup time	Minutes; no fiducials or complex calibration needed	Typically 15–30 minutes; requires fiducials and calibration
Ease of use	Simple workflow; intuitive manual alignment; minimal training needed	Complex workflow; requires professional staff and technical training
Learning curve	Short; stable performance achieved after brief practice on a simulated model	Long; requires extensive training and repeated practice
Accuracy	Mean error ~3–4 mm in this proof-of-concept study	Millimeter or sub-millimeter accuracy; extensively validated
Hardware needs	Standard Android smartphone/tablet with camera and display	Specialized navigation hardware and optical tracking systems
Clinical status	Proof-of-concept; currently used for preoperative localization only	Established; widely adopted for intraoperative navigation in neurosurgery

1. Image Construction Using 3D Slicer **(refer to Video 1)**

Step 1: Acquire the imaging data sequence from the hospital PACS system. The most common format is DICOM, but 3D Slicer also supports other formats such as NRRD and Nifty.

Step 2: Import the data into 3D Slicer and open the Segment Editor module for image segmentation.

Step 3: Use the Threshold tool to segment the skin, and reconstruct a solid head model.

Step 4: Reconstruct the lesion using the Paint or other suitable tools. According to the lesion location, use the Draw Tube tool to delineate the surgical trajectory.

Step 5: Adjust the 3D view so that it is perpendicular to the planned trajectory, and set the skin layer to semi-transparency.

Step 6: On the semi-transparent skin surface, outline the projected boundary of the lesion, and then switch the skin back to opaque.

Step 7: Capture the current 3D view by screenshot or by photographing the computer screen with a smartphone. The resulting image is then used as the reference for NP-Guide localization.

2. Practical Tips for NP-Guide

- **Tip 1:** When creating the projection image, orient the image plane as perpendicular as possible to the planned surgical approach.
- **Tip 2:** During projection-based localization, using a portable stand to fix the device provides more stable results.
- **Tip 3:** Drawing the lesion boundary on the skin surface and keeping the skin opaque yields better visualization than making the skin semi-transparent to display the lesion directly.

3. NP-Guide Startup Workflow

- **Step 1:** Download the APK file from GitHub (https://github.com/xmszj/NP-Guide) and install it on a mobile device.
- **Step 2:** Open the mobile device and launch NP-Guide as you would with any regular app.
- **Step 3:** The main interface of NP-Guide provides function descriptions in both Chinese and English.
- **Step 4:** The interface includes several buttons:

- Gallery: Opens the local gallery and imports images into the app.
- Mirror: Mirrors the current image horizontally, useful for projecting unprocessed raw images.
- Exit: Closes the program.
- Rotate and Opacity: Used to adjust the orientation and transparency of the current image (see **Figure 2**).

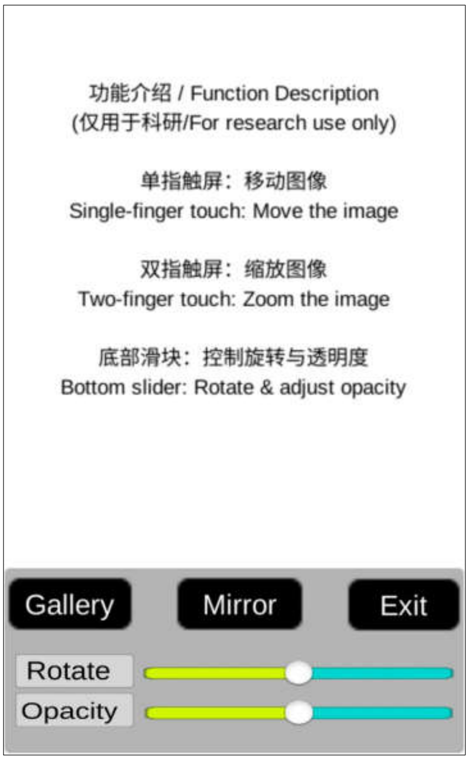

Figure 2. Main interface of NP-Guide.

4. Image Data Processing

Two approaches are available for preparing images suitable for NP-Guide.

4.1 Approach 1: 3D Image Modeling

When time permits, patient imaging data can be processed using medical image modeling software. In this supplementary material, we demonstrate the process with 3D Slicer as an example. The scalp contour can be quickly reconstructed using the thresholding tool, while the lesion can be delineated directly with the paint tool. The entire process can be completed in approximately 1 minute (**Figure 3**).

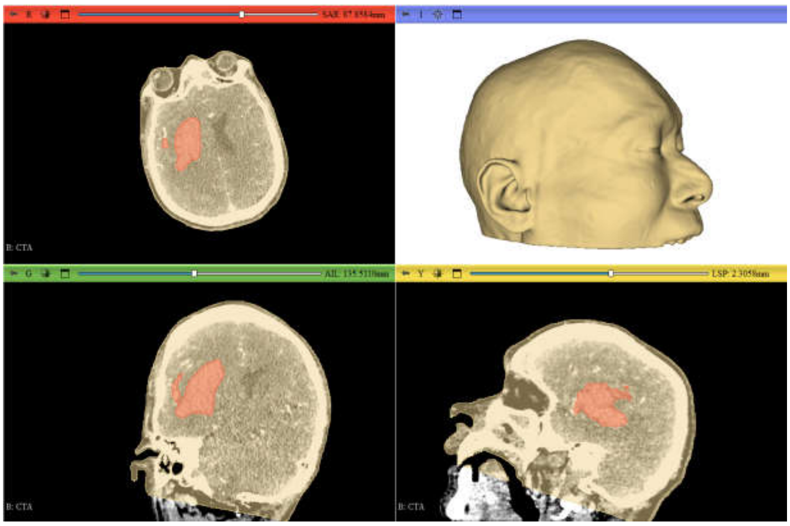

Figure 3. 3D reconstruction of the scalp and lesion using the Threshold and Paint tools in 3D Slicer.

4.1.1 View Adjustment

Adjust the transparency of the scalp model to make the lesion visible (**Figure 4A**).

At this stage, the surgeon should design the skin incision based on the spatial relationship between the lesion and the scalp. Since the lesion can be projected onto the scalp from different viewing angles, 2D images can be used as a reference during incision planning. The principle is to choose the shortest path while avoiding major vessels and nerves (**Figure 4B–D**).

It is important to ensure that the surgical approach is perpendicular to the screen (**Figure 4D**), which can then be used as the projection image for localization.

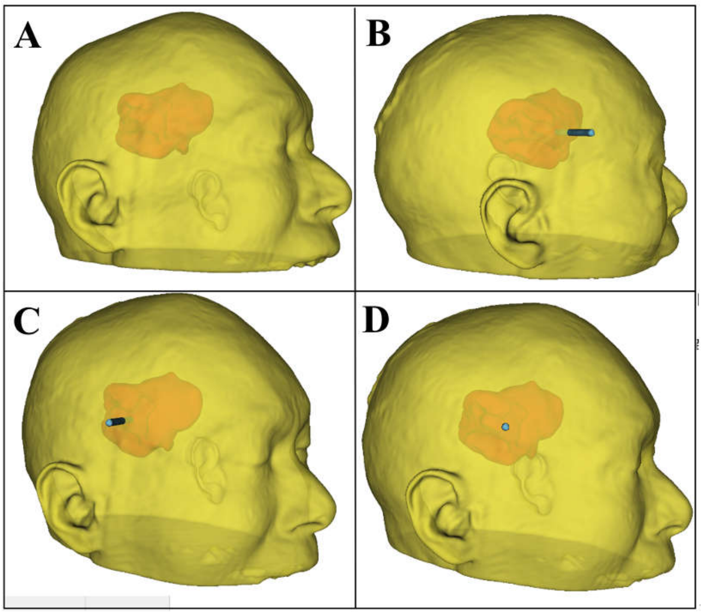

Figure 4. Viewing angles of the reconstructed images. (A) Adjusting scalp model transparency to visualize the lesion. **(B–C)** The lesion remains visible from different viewing angles. **(D)** Correct viewing angle, where the surgical approach is perpendicular to the display.

4.1.2 Enhanced Localization

For improved localization accuracy, the lesion boundary can be delineated on the basis of the view shown in Figure 3D (**Figure 5A**). The scalp model is then set back to opaque (**Figure 5B**) and used as the projection image. Direct projection with this image can provide more accurate localization results (**Figure 5C**).

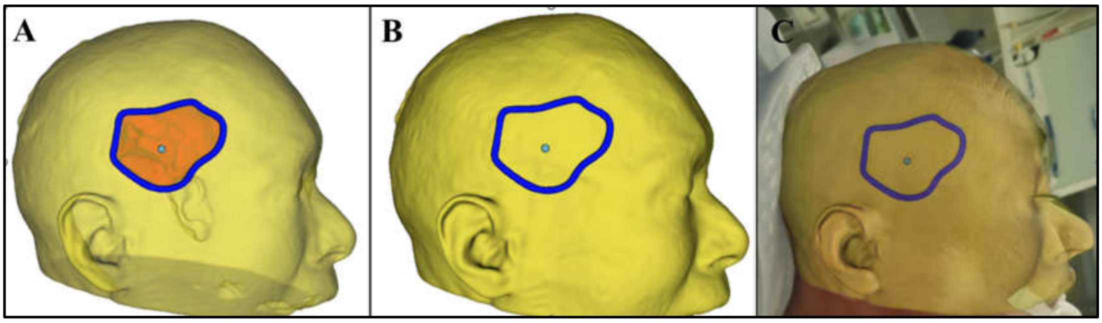

Figure 5. Projection method with delineated lesion boundary. (A-B) Lesion boundary delineated on the scalp surface. (C) Final projection image used for accurate localization.

4.2 Approach 2: Projection Localization Using the Largest Imaging Plane

4.2.1 The lesion is located near the midline, close to the sagittal sinus

Step 1: Using the patient's axial imaging series, identify the slice containing the lesion and place the mouse pointer at the lesion center (**Figure 6A**, **yellow arrow**).

Step 2: Without moving the pointer, scroll through the slices with the mouse wheel until the largest cross-sectional plane of the lesion is reached (e.g., at the skull base or brainstem level) (**Figure 6B**).

Step 3: Capture the selected slice as a JPEG image by photographing the computer screen with a smartphone running NP-Guide. Import the image into NP-Guide, select it from the gallery, and apply the *Mirror* function to obtain a mirrored image (**Figure 6C**). *Note: mirror mode must be applied for right-sided lesions to ensure correct anatomical orientation during projection.*

Step 4: Adjust image transparency with the slider, and use rotation, scaling, and translation functions to overlap the projected image with the patient's scalp contour. The position corresponding to the mouse pointer on the image then represents the lesion projection point on the patient's skin (**Figure 6D**).

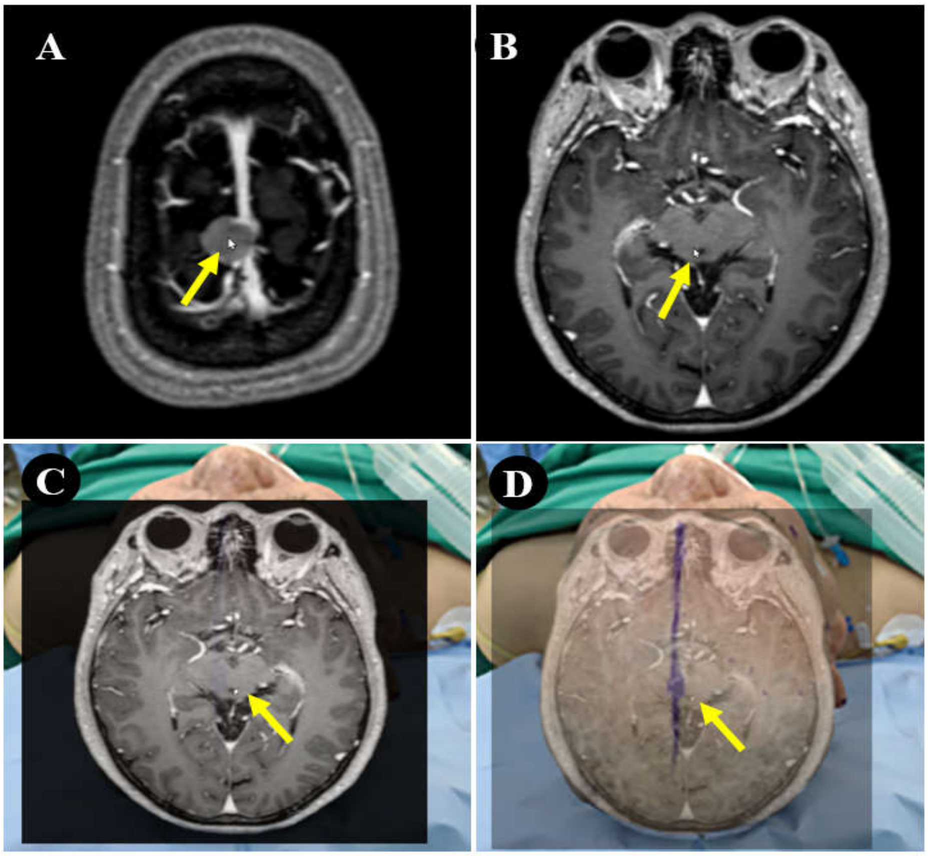

Figure 6. Projection-based localization using the axial plane. (A–B) Selection of the largest axial plane with the tumor center indicated by the mouse pointer (yellow arrow). (C–D) Mirrored projection image for matching and localization when the lesion is located on the right side.

4.2.2 The lesion is located in the basal ganglia or temporal lobe

Step 1: Using the sagittal imaging series, identify the slice that passes through the lesion in the basal ganglia and place the mouse pointer at the lesion center (**Figure 7A, red arrow**).

Step 2: Without moving the pointer, scroll through adjacent sagittal slices until the slice showing the lesion at its largest extent is obtained (**Figure 7B**).

Step 3: Capture this slice as a JPEG image by photographing the computer screen with a smartphone running NP-Guide. Import the image into NP-Guide, select it from the gallery (**Figure 7C**).

Step 4: Adjust image transparency with the slider, and use rotation, scaling, and translation functions to overlap the projected image with the patient's scalp contour in the sagittal orientation. The point corresponding to the mouse pointer then represents the scalp projection of the basal ganglia lesion(**Figure 7D**).

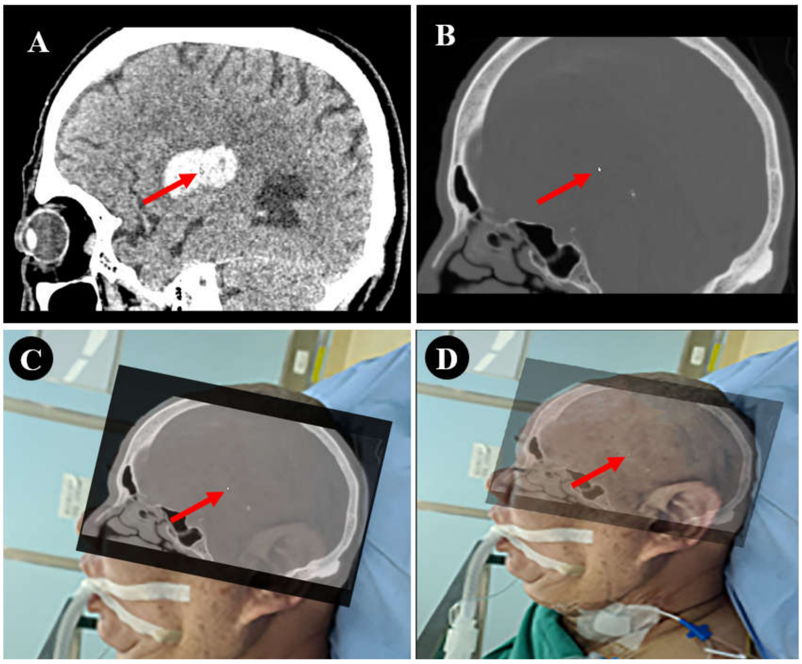

Figure 7. Projection-based localization using the sagittal plane. The largest sagittal plane is selected for projection when the lesion is located in the basal ganglia or temporal lobe.

5. Workflow Clarifications for NP-Guide

The following section provides detailed clarifications regarding the workflow of NP-Guide.

Q1. After segmentation in 3D Slicer, how are images exported to the smartphone?

Two approaches are commonly used:

1. Taking screenshots on the computer and transferring them to the smartphone via a USB data cable.
2. Capturing the computer screen directly using the smartphone camera.

Q2. Which images should be exported, and in what format?

All required images are obtained either by screenshots or by photographs of the computer screen. The formats are JPEG or PNG.

Q3. Does the software handle multiple formats (Dicom, Nifti, JPEG, PNG)?

The data-processing software supports Dicom, Nifti, JPEG, and PNG formats. However, NP-Guide can only read formats supported by the smartphone's internal gallery, namely JPEG and PNG.
For Dicom or Nifti data, images can be opened on a computer and then converted to JPEG/PNG via screenshots or photographs.

Q4. How are segmented masks exported?

NP-Guide currently reads only JPEG and PNG images; therefore, exporting segmented masks is not required.

Q5. How many segmentations can be used simultaneously (skin + lesion only, or also skull, cortical surface, vascular structures)?

The number of segmentations depends on the surgeon's needs. For projection-based localization, skin and lesion are mandatory. Other structures (e.g., skull, cortical surface, vessels) may be optionally included. For example, if vessel projection is of interest, images can be generated as shown in Video 1.

Q6. Can multiple image sets (CT, MRI, vascular images) be merged? What is the workflow for visualizing different layers in the app?

Currently, NP-Guide supports only single 2D images and does not allow multi-modal fusion. However, multimodal fusion can be performed on a computer prior to export. The processed composite image can then

be saved as a JPEG for use in NP-Guide. This process may increase preparation time, and since NP-Guide serves as a coarse, auxiliary localization tool, complex multimodal processing is not generally recommended.

Q7. If a 3D atlas segmentation (e.g., basal ganglia) is fused into the patient's imaging, can it be visualized in the patient's space?

Yes. In such cases, the skin layer should be set to semi-transparent to allow visualization of deeper structures (e.g., basal ganglia). Although primarily intended for preoperative localization, this feature may also be valuable for teaching and physician–patient communication.

Q8. How are images imported into the smartphone and the app?

Once images are transferred to the smartphone memory, NP-Guide can open them directly. Launch the app, tap **Gallery,** and select the desired image from the smartphone album.

6. Summary

The recommendations described above are intended as practical references for improving the use of NP-Guide. Users are encouraged to further explore additional techniques and optimize workflows based on their own clinical experience. NP-Guide is designed as an adjunctive tool to support medical localization, offering flexibility, portability, and ease of use. With continued feedback and refinement, it has the potential to further expand its role in neurosurgical navigation and other medical applications.
